# Validated HPTLC and antioxidant activities for quality control of catechin in a fermented tea (*Camellia sinensis* var. assamica)

**DOI:** 10.1002/fsn3.2285

**Published:** 2021-05-04

**Authors:** Phanit Thammarat, Sasithorn Sirilun, Rungsinee Phongpradist, Araya Raiwa, Hataichanok Pandith, Jutamas Jiaranaikulwanitch

**Affiliations:** ^1^ Department of Pharmaceutical Sciences Faculty of Pharmacy Chiang Mai University Chiang Mai Thailand; ^2^ Innovation Center for Holistic Health, Nutraceuticals, and Cosmeceuticals Faculty of Pharmacy Chiang Mai University Chiang Mai Thailand; ^3^ Department of Biology Faculty of Sciences Chiang Mai University Chiang Mai Thailand; ^4^ Research Center in Bioresources for Agriculture, Industry and Medicine Faculty of Science Chiang Mai University Chiang Mai Thailand

**Keywords:** antioxidant, *Camellia*, catechin, fermented tea, HPTLC, miang

## Abstract

Miang, a Thai traditional fermented tea (*Camellia sinensis* var. assamica), is exploited as nutraceutical and cosmeceutical ingredients despite limited standardization studies. Thus, this research aimed to develop a simple and rapid method for miang quality control using catechin and high‐performance thin‐layer chromatography (HPTLC) validated according to the International Council for Harmonisation of Technical Requirements for Pharmaceuticals for Human Use (ICH) and the Association of Official Analytical Collaboration (AOAC). The developing solvent consisting of toluene: ethyl acetate: acetone: formic acid (6:6:6:1 v/v/v/v) showed acceptable specificity with *R*_f_ value of 0.54 ± 0.02 and linearity with correlation coefficient of 0.9951. The recovery was 98.84%–103.53%, and the RSD of intra‐ and inter‐day precision was 0.70%–3.00% and 1.93%–4.94%, respectively. Miang ethyl acetate fraction is suggested to be attractive ingredient due to rich catechin (25.78 ± 0.53%), prolonged stability at 40 ^◦^C, and strong antioxidants determined by the assays of ABTS (IC_50_ = 3.32 ± 0.74 mg/ml), FRAP (89.05 ± 15.49 mg equivalent of FeSO_4_/g), and inhibition of lipid peroxidation (IC_50_ = 4.36 ± 0.67 mg/ml).

## Graphical abstract

The combination of validated HPTLC method and simple antioxidant assays provides a rapid and cost‐effective approach for fermented tea standardization.

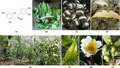

## 1 INTRODUCTION

**FIGURE 1:**
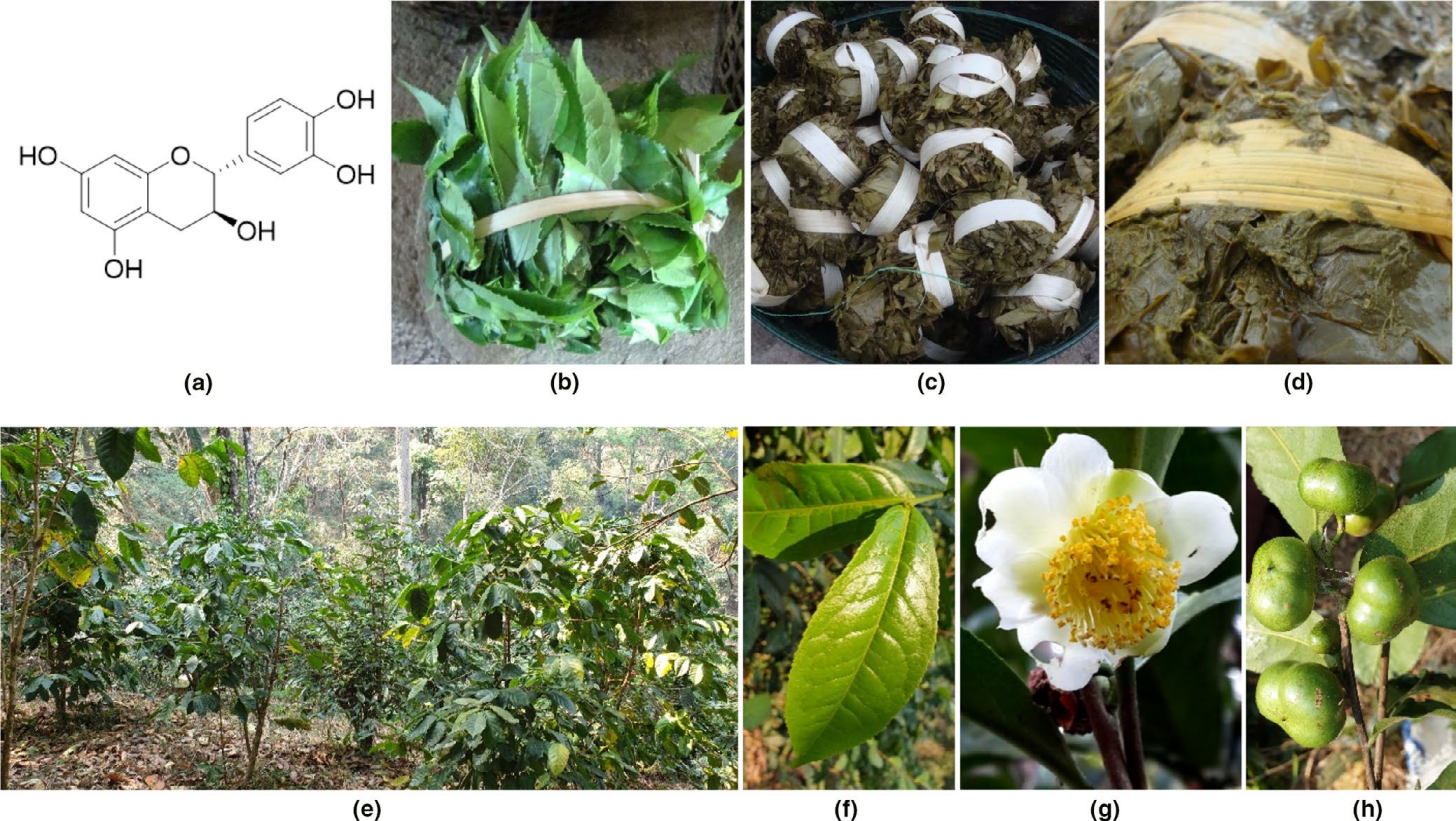
*Camellia sinensis*; (a) a structure of (+)‐catechin; (b) a preparation of collected fresh leaves for a fermentation process; (c and d) appearances of traditional fermented tea leaves, locally known as miang; (e) cultivated fields around the highland areas of northern Thailand; (f) leaves; (g) flower; and (h) fruits.

Tea (*Camellia sinensis*) is one of the most consumed beverages. Moreover, tea extracts are popular ingredients in nutraceuticals, herbal supplements, and cosmeceuticals. Some health applications include diet‐induced obesity suppression (Murase et al., 2002), cardiovascular disease protection (Nagao et al., 2007), tumor progress prevention (Singh et al., 2011), immune modulation (Marinovic et al., 2015; Matsunaga et al., 2001), and infection prevention and treatments (Matsumoto et al., 2011; Tran, 2013). Tea catechins (Figure 1) are potent antioxidants (Grzesik et al., 2018; Higdon & Frei, 2003). In Europe and the United States, tea and pome are the main catechin sources (Chun et al., 2007; Vogiatzoglou et al., 2015). Catechins induce antioxidant activities via scavenging free radicals, chelating metal ions, inhibiting pro‐oxidant enzymes, stimulating antioxidant enzymes, and producing phase II detoxification enzymes and antioxidant enzyme (Bernatoniene & Kopustinskiene, 2018; Higdon & Frei, 2003; Youn et al., 2006). Catechin accumulation is influenced by plant varieties, soil compositions, weather conditions, harvesting season, storage conditions, and product processing methods (Council of Europe, 2018; Zuo et al., 2002). Therefore, Good Manufacturing Practice (GMP) starting from raw materials to final products is essential to achieving highest health benefits. GMP is also used to assure extraction efficiency and proper storage conditions (Mukherjee, 2002).

In northern Thailand, popular ethnic tea is prepared from *C. sinensis* var. assamica by special fermentation, producing tea locally known as “miang” (Figure 1) (Tanasupawat et al., 2007; Unban et al., 2019). Briefly, tea leaves are steamed 1–2 hr, then fermented under anaerobic condition 1–4 weeks to produce “miang‐faat” (astringent taste) or three to twelve months to produce “miang‐som” (sour taste) (Unban et al., 2019). Miang is not a beverage but a chewing tea consumed as snacks, either alone, with salt, or with other condiments such as roasted coconut, garlic, and shredded ginger (Khanongnuch et al., 2017). It is traditionally often served at religious gatherings and funerals (Khanongnuch et al., 2017). Recently, miang has gained increasing interests due to its antioxidants, antimicrobials, and health‐promoting probiotics (Chaikaew et al., 2017; Klayraung & Okonogi, 2009; Tanasupawat et al., 2007; Unban et al., 2019). Therefore, it could commercially be developed. While most studies have been focused on miang bioactivities (Chaikaew et al., 2017; Khanongnuch et al., 2017; Klayraung & Okonogi, 2009; Tanasupawat et al., 2007; Unban et al., 2019), miang standardization and quality control have not yet been investigated. *C. sinensis* possess rich phenolics of which 85% are catechins such as (‐)‐epigallocatechin 3‐gallate (EGCG), (‐)‐epigallocatechin (EGC), (‐)‐epicatechin 3‐gallate (ECG), (‐)‐epicatechin (EC), and (+)‐catechin (C) (Wang et al., 2000). Similarly, to other studies (Zuo et al., 2002), miang total phenolics also vary by plantation area, tea plucking, and ages of tea leaves (Khanongnuch et al., 2017). Owning to health benefits, catechins are used in tea standardization; for example, green tea is standardized by EGCG (min 8%) and caffeine (min 1.5%) according to European Pharmacopoeia (9th ed, 2018) (Council of Europe, 2018). Since tea GMP substantially relies on catechin determination, developing a validated analytical method is an essential prerequisite to miang quality control. Method validation is the process following guidelines such as ICH and AOAC to assess quality, reliability, and consistency of analytical procedure. Additionally, a validated analytical method should be versatile, rapid, and cost‐efficient.

Catechin analyses are mostly developed using liquid chromatography and capillary electrophoresis techniques (Dalluge & Nelson, 2000). However, high‐performance thin‐layer chromatography (HPTLC) is a simple and highly sensitive technology, is proven to determine various phytoconstituents, and features low solvent consumption, minimal sample preparation, and concurrent analysis of high throughput assays with minimal costs (Upton, 2010). HPTLC is added to many natural product monographs and regulations worldwide, including European Pharmacopoeia (Council of Europe, 2018) and American Herbal Pharmacopoeia (Upton, 2010). It also provides fingerprints to plant species and their origin (Upton, 2010; Council of Europe, 2018). Therefore, HPTLC is not only useful for a quantitative but also qualification controls of natural products. Several applications include authentication, adulteration assessment, and phytochemical monitoring during extraction. Additionally, HPTLC empowers automation, scanning, condition optimization, detection, and minimum sample requirement (Attimarad et al., 2011). So far, HPTLC has not yet been developed for miang. Therefore, in this research, ICH and AOAC guidelines (AOAC International, 2016; ICH, 1994, 2003) were applied to create a validated HPTLC of catechin for miang quality control. Moreover, simple antioxidant assays were included to test association between antioxidant activities and catechin. The evaluations of extraction and storage condition were also presented.

## 2 MATERIALS AND METHODS

### 2.1 Chemicals and reagents

All chemicals and solvents were analytical grade. Standard catechin was purchased from Sigma^®^ (Sigma‐Aldrich, USA). Stock solutions of standard catechin were freshly prepared for daily use. Stationary phase was TLC plates precoated with silica gel 60 F_254_ (20x10 cm) and thickness of 0.2 mm purchased from Merck (USA). Ethanol, hexane, dichloromethane, ethyl acetate, and methanol were purchased from Labscan (Dublin, Ireland). 2,20‐azino‐bis‐3‐ethylbenzothiazoline‐6‐sulfonic acid (ABTS), 2,4,6 tripyridyl‐s‐triazine (TPTZ), 6‐hydroxy‐2,5,7,8‐tetramethylchroman‐2‐carboxylic acid (Trolox), and linoleic acid were purchased from Sigma‐Aldrich (St. Louis, MO, USA). Tris base was purchased from Fisher Chem Alert (Fair Lawn, NJ, USA). Potassium persulfate, ferric chloride, ferrous sulfate, and sodium acetate were purchased from Fisher Chemicals (Loughborough, UK). Hydrochloric acid, acetic acid, trichloroacetic acid, thiobarbituric acid, and ascorbic acid were purchased from Merck (Darmstadt, Germany).

### 2.2 Plant material and sample preparation

**FIGURE 2:**
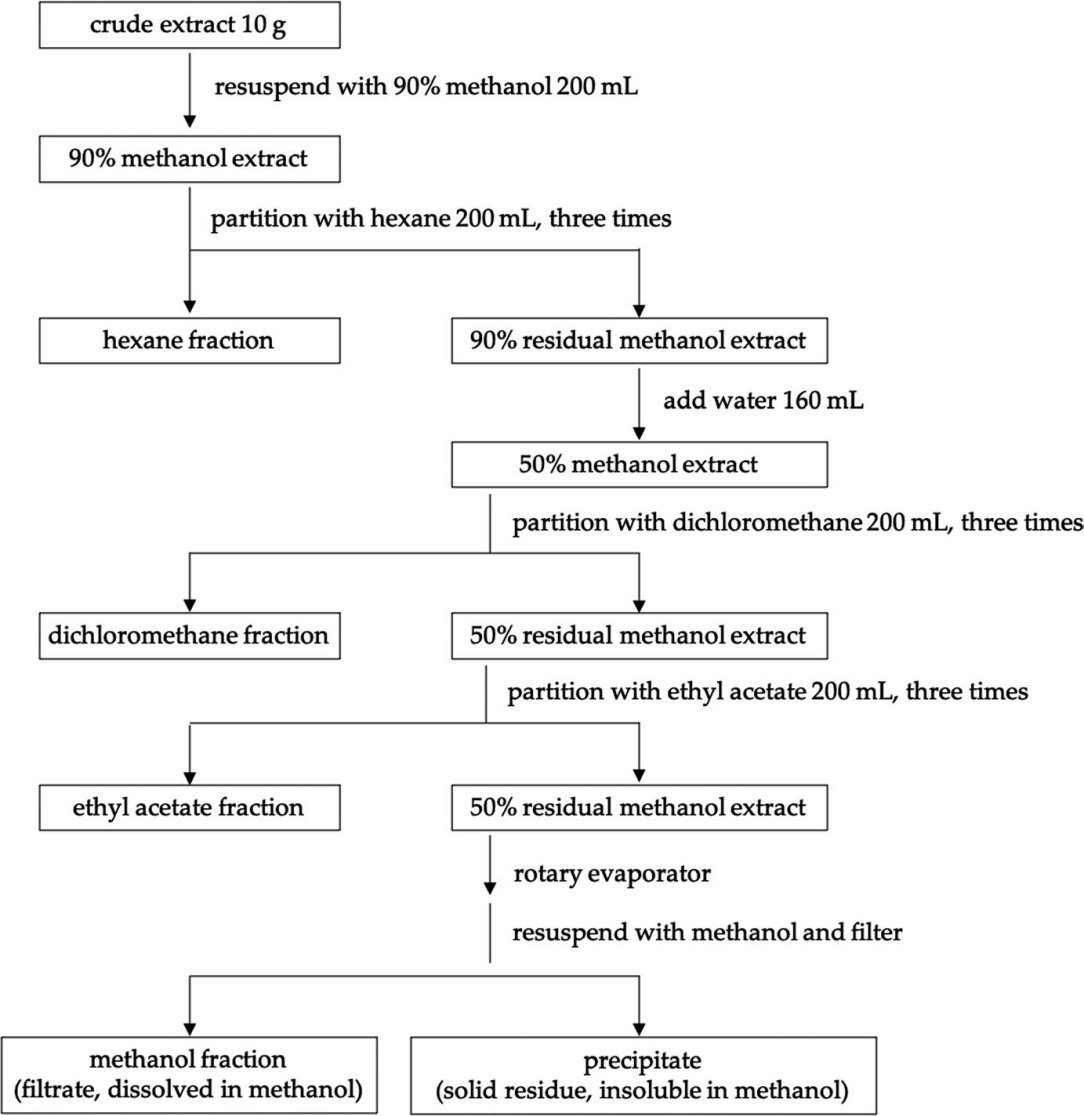
Solvent–solvent partitioning of miang ethanolic crude extract to obtain semi‐purified fractions of hexane, dichloromethane, ethyl acetate, and methanol.

Miang samples were obtained from and authenticated by the Highland Research and Development Institute, Chiang Mai, Thailand (voucher number 0023247, Faculty of Pharmacy, Chiang Mai university, Thailand). Raw materials were dried (50°C for 8 hr), then ground and macerated in 95% ethanol (1 kg dried plant per 10 L 95% ethanol) for 72 hr. The macerated extracts were filtered and dried using a rotary evaporator to produce crude extract (CE). CE was then sequentially partitioned by polarity into four semi‐purified fractions of hexane (HF), dichloromethane (DF), ethyl acetate (EF), and methanol (MF) (Figure 2) (Emran et al., 2015). Briefly, for the fractionated samples, crude extract (10 g) was re‐dissolved with 90% methanol and then partitioned with hexane three times. After that, distilled water (160 ml) was added to the 90% residual methanol extract to obtain the 50% methanol extract. This step increased the extract volume and polarity prior to being further partitioned by dichloromethane and ethyl acetate, respectively. The final 50% residual methanol extract was subjected to a rotary evaporator to remove solvent, then re‐dissolved with methanol and filtered to isolate precipitate and methanol fraction. The process was carried out in a separatory funnel at room temperature. With each solvent, the partitioning was repeated three times from the residual extract in the separatory funnel. Then, all three partitions of each solvent were pooled, dried with NaSO_4_, then filtered, and subjected to a rotary evaporator to remove solvent under a reduced pressure to obtain a semi‐purified fraction. The solvent–solvent partitioning experiment was performed in triplicate and percent yield of semi‐purified fraction was calculated.

### 2.3 Standard solutions

Catechin stock solution was prepared at 1 μg/μl in methanol. The working standard solutions were prepared by diluting the stock solution with methanol to 0.25, 0.50, 0.60, and 0.80 μg/μl.

### 2.4 Chromatographic conditions

Chromatographic separation was achieved on HPTLC plates (20 × 10 cm), precoated with silica gel 60 F_254_ and thickness of 0.2 mm with aluminum sheet support. Solutions of standard catechin and miang extracts were applied as bands (8.0 mm width) along one edge of the plate. Samples were loaded and developed on the same chromatographic plate. Sample loadings were carried out by a Camag (Muttenz, Switzerland) Linomat 5 sample applicator equipped with a 100 μl Hamilton syringe. Ascending development to a distance of 80 mm was performed after the plate was saturated in developing solvent (toluene: ethyl acetate: acetone: formic acid (6:6:6:1 v/v/v/v)) for 20 min at room temperature (25 ± 5°C) with a relative humidity of 47%. After completing the development, plates were dried and then measured at 254 nm by a Camag TLC Scanner 4 using the deuterium lamp and WinCAT software. The slit dimension was 8 x 0.2 mm with scanning speed of 40 mm/s.

### 2.5 Method validations

The HPTLC method was assessed the validation for the following parameters.

#### 2.5.1 Specificity

Specificity was determined by overlaying the spectrums of standard catechin and CE and comparing *R*_f_ values.

#### 2.5.2 Linearity

10 µl of catechin solutions (0.25, 0.50, 0.60, 0.80, and 1.00 µg/µl) was applied to HPTLC plates to obtain a calibration curve. Linear regression was plotted between peak heights and amount of standard catechin. The correlation coefficient (*r*), slope, and intercept were determined.

#### 2.5.3 Accuracy

Accuracy was ascertained by measuring percent recovery of a known amount of standard catechin spiked to crude extract. In the optimization process, catechin target content was predetermined by using crude extract (4 µg/µl) with an injection volume of 10 µl. According to ICH guideline, an accuracy is suggested to be tested on the contents covering 80%–120% of target content (ICH, 1994). Therefore, in this research, the amounts spiked were selected to cover 80%, 100%, and 120% of the preliminary catechin target quantity, each performed in triplicate. These solutions were finally adjusted to a concentration similarly to CE at 4 µg/µl. In each sample, a volume of 10 µl was applied to HPTLC plates to analyze catechin. Percent recovery was calculated using the following equation:

$$\%\;\text{recovery}=\left(\text{concentration found}/\text{concentration added}\right)\times 100.$$

#### 2.5.4 Precision

Precision was computed as percent relative standard deviation (% RSD) for the assays performed on the same day (intra‐day) and across three consecutive days (inter‐day) by using catechin standard solutions of 0.25, 0.50, and 1.00 µg/µl, each performed with five replicates. The volume of 10 µl was applied to HPTLC to analyze catechin. The precision parameter was expressed as % RSD by measuring the peak height.

#### 2.5.5 Limit of detection and limit of quantification

Sensitivity was determined with respect to limit of detection (LOD) and limit of quantification (LOQ) of the catechin content. Both parameters were computed from six multiple sets of regression coefficients using the formula k*SD of intercept/mean of slope, where *k* = 3.3 for LOD and 10 for LOQ.

### 2.6 Determination of catechin content in miang extracts

In the optimization process, CE’s working solution (4 µg/µl) showed a peak height in the validated range. Thus, all fractions were evaluated using preliminary working concentrations of 4 µg/µl. However, EF showed peak height exceeding the standard curve range. Therefore, CE and EF’s stock solutions were prepared at 20 µg/µl which were then diluted to obtain working solutions at 4 and 2 µg/µl, respectively. On the other hand, HF, DF, and MF (4 µg/µl) showed lower peak heights than the standard curve range. Thus, the working solutions of HF, DF, and MF were directly prepared at 32 μg/μL. For each sample, a volume of 10 µl was applied to HPTLC plates to analyze catechin. The experiment was performed in triplicate for each sample solution.

### 2.7 Stability study of catechin contents in miang crude extract and ethyl acetate fraction

CE and EF were kept at 4 and 40°C to study stability profiles. Catechin was determined at 0‐, 3‐, and 6‐month periods (ICH, 2003). Stock solutions of CE and EF were prepared at 20 µg/µl. The working solutions were prepared by diluting stock solution to either 4 µg/µl or 8 µg/µl, depending on the observed catechin peak height. For each sample, a volume of 10 µl was applied to HPTLC plates to analyze catechin. The experiment was performed in triplicate for each sample.

### 2.8 Determination of antioxidant activities

Miang extracts were investigated for antioxidant activities by the following assays.

#### 2.8.1 2,20‐azino‐bis‐3‐ethylbenzothiazoline‐6‐sulfonic acid (ABTS) assay

Miang extracts were tested for free radical scavenging activities by ABTS assay. Briefly, ABTS^•+^ radical cations were generated by mixing 7 mM ABTS solution with 2.5 mM potassium persulfate at a ratio of 1:1. The ABTS^•+^ solution was diluted with phosphate buffer to a working solution which had an absorbance of 0.700 ± 0.02 at 734 nm. The ABTS^•+^ working solution (0.9 ml) was reacted with each sample (0.1 ml) at different concentrations for 3 min. The absorbance was measured at 734 nm. Trolox was used as a standard and the ABTS^•+^ scavenging activity of each sample was expressed as Trolox equivalent antioxidant capacity (TEAC) which was calculated from the calibration curve of Trolox antioxidant activity (Saenjum et al., 2010).

#### 2.8.2 Ferric reducing antioxidant power (FRAP) assay

Miang extracts were tested for total reducing power by FRAP assay. Briefly, FRAP reagent was prepared by mixing acetate buffer, 2,4,6 tripyridyl‐s‐triazine (TPTZ) and FeCl_3_.6H_2_O in a ratio of 10:1:1. The mixture was freshly prepared and protected from light. The antioxidant activity of test samples was evaluated by mixing 1,800 µl of FRAP reagent, 60 µl of test sample, and 180 µl of deionized water. The absorbance of the reaction mixture was measured at 593 nm. Ferrous sulfate (FeSO_4_) was used as a standard and the ferric ions reducing power of each sample was expressed as FeSO_4_ equivalent which was calculated from the calibration curve of ferrous sulfate reducing power activity (Benzie & Strain, 1996).

#### 2.8.3 Inhibition of lipid peroxidation assay

Miang extracts were tested for inhibitory capacities against lipid peroxidation by a method modified from Frankel and Neff (1983). Briefly, each extract was evaluated by mixing 100 µl of extract solution (1 mg/ml), 500 µl of linoleic acid (20 mM), 30 µl of Tris HCl (100 mM, pH 7.5) and 10 µl of ascorbic acid (20 mM). The reaction mixture was then activated by adding 10 µl of Fe_2_SO_4_.7H_2_O (40 mM) and incubating at 37°C for 30 min. The termination of the reaction was conducted by adding 104 µl of 40% v/v trichloroacetic acid and 200 µl of thiobarbituric acid (1% w/v) and incubating at 95°C for 10 min. Absorbance of the mixture reaction was then measured at 532 nm by using an ultraviolet–visible (UV–visible) spectrophotometer (Beckman Coulter DTX880, Fullerton, CA, USA). The lipid peroxidation inhibitory activity of each extract was expressed as percent inhibition and half maximal inhibitory concentration (IC_50_). Percent inhibitory activity was calculated using the following equation:

%inhibition=[(A‐B)/A]×100,

where *A* is the absorbance of control reaction with the absence of miang extract and *B* is absorbance of sample reaction with the presence of miang extract. The extract concentration that causes 50% inhibition against lipid peroxidation or IC_50_ value was determined from the curve derived from the graph plotted between percent inhibitions against extract concentrations (10, 25, 50, 100, 200, 400, 600, and 1,000 µg/ml) using GraphPad Prism v8.0 (GraphPad, La Jolla, CA, USA). All determinations were carried out in triplicate. Lower IC_50_ value indicates higher antioxidant activity.

### 2.9 Statistical analysis

All data are presented as mean ± standard deviation (*SD*). Significant differences were assessed by either *t* test or one‐way ANOVA followed by post hoc tests. All statistical analyses and graphical presentation were carried out on GraphPad Prism v8.0 (GraphPad, La Jolla, CA, USA) and SPSS 17.0 (Chicago, USA).

## 3 RESULTS AND DISCUSSION

### 3.1 Miang extracts

**FIGURE 3:**
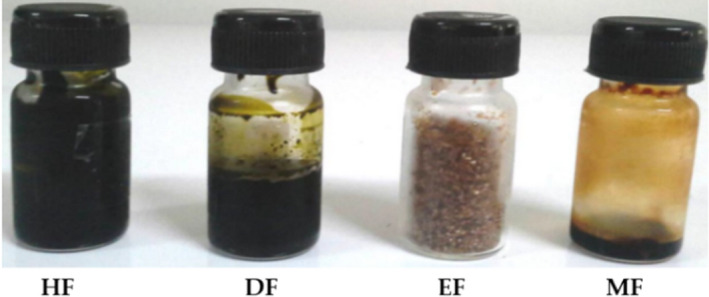
Physical appearances of miang HF (hexane fraction), DF (dichloromethane fraction), EF (ethyl acetate fraction), and MF (methanol fraction).

**FIGURE 4:**
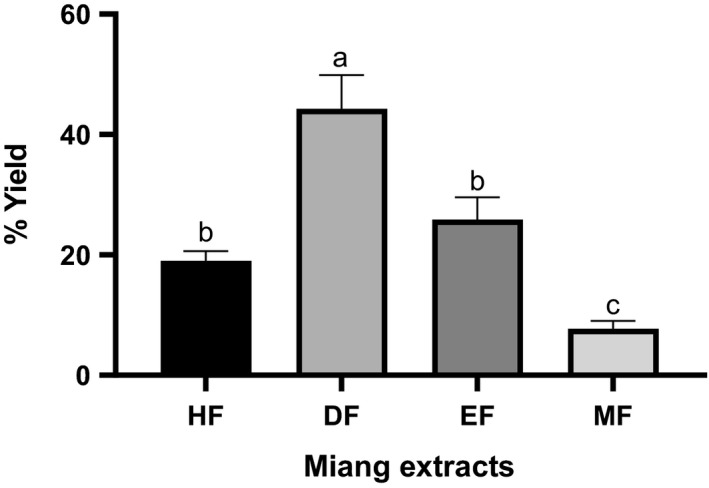
Extractable yields of miang HF (hexane fraction), DF (dichloromethane fraction), EF (ethyl acetate fraction), and MF (methanol fraction). Percent yield is a ratio of fraction weight and crude extract weight. All data are presented as mean ± *SD* of three independent experiments. Letters (a, b, and c) indicate significant differences of means between the groups based on Tukey's HSD one‐way ANOVA (*p* <.05).

CE was prepared by macerating dried leaves in 95% ethanol and then sequentially partitioned by polarities to produce fractions HF, DF, EF, and MF. Extracts’ yields and physical appearances were distinct among fractions (Figures 3 and 4). DF had the highest yield (44.48 ± 5.63%). On the contrary, MF showed the lowest yield (7.61 ± 1.30%). HF and EF showed the comparable yields which were 19.03 ± 1.57% and 26.09 ± 3.73%, respectively. CE and HF were both blackish greens, highly viscous, and liquid. DF was dark green, highly viscous, and liquid. EF was golden‐reddish brown, coarse sand‐like, and semi‐solid. MF was dark brown, viscous, and liquid. Taken together, this might suggest that solvent–solvent partitioning was able to isolate semi‐purified fractions from CE, depending on the constituent polarity indices.

### 3.2 HPTLC method validation

HPTLC for catechin analysis in miang extracts was conducted using a developing solvent consisting of toluene: ethyl acetate: acetone: formic acid (6:6:6:1 v/v/v/v). Method validation was performed according to ICH and AOAC. Each of validating parameters is presented in the following results.

#### 3.2.1 Specificity

**FIGURE 5:**
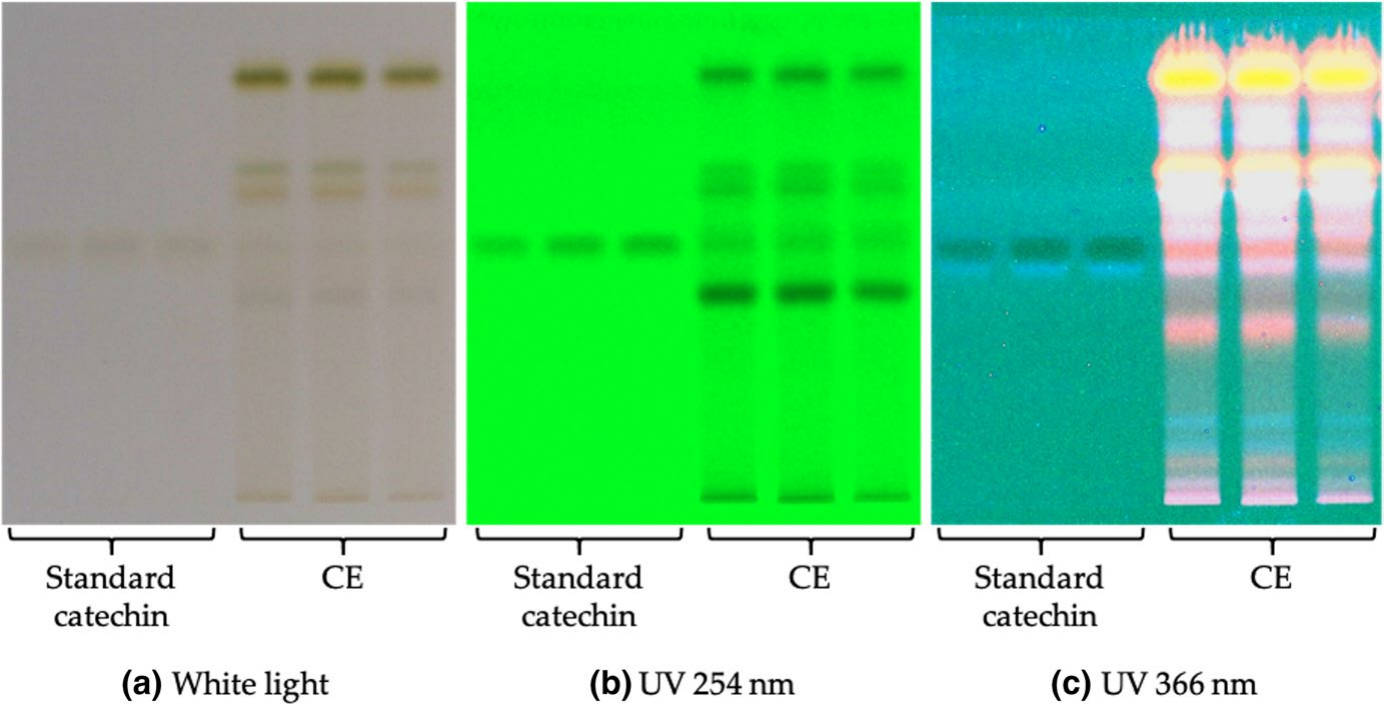
High‐performance thin‐layer chromatography (HPTLC) profiles of standard catechin and miang crude extract (CE) by using a developing solvent consisting of toluene: ethyl acetate: acetone: formic acid (6:6:6:1 v/v/v/v) and measuring at; (a) white light; (b) UV 254 nm; and (c) UV 366 nm.

Specificity is ability to assess analyte in a presence of components that may be expected in the sample matrix. Method specificity of HPTLC was normally obtained by comparing retardation factor (*R*_f_) of standard analyte. *R*_f_ value of analyte should not be interfered with other substances in the sample matrix (Alam et al., 2020; Alqarni et al., 2021; Foudah et al., 2020). Peak purity was evaluated by comparing the spectrums of standard catechin with CE sample. Figure 5 shows that the *R*_f_ of standard catechin was not interfered by other bands presenting in CE and was detected at 254 nm. The HPTLC produced a moderate resolution and sharp peak of standard catechin with *R*_f_ value of 0.54 ± 0.02.

#### 3.2.2 Linearity

**FIGURE 6:**
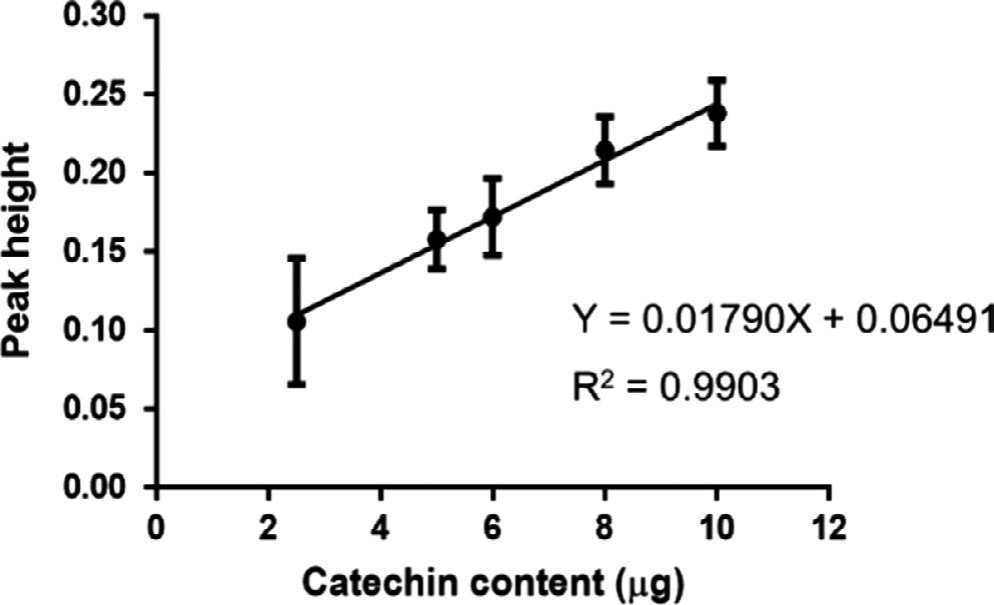
Calibration curve of standard catechin from 2.5 to 10 µg. Data are presented as mean ± *SD* of triplicate.

Linearity is ability (within a given range) to obtain test results which are directly proportional to concentration of analyte in the sample. It was determined by plotting a graph of peak height versus standard content to obtain a correlation coefficient (*R* ^2^) and equation of the line. Figure 6 shows a linear response of catechin content over the range of 2.5 and 10 µg with a good fit indicated by large *R* ^2^ value of .9903 (*r* = .9951). The linear regression has an equation of *y* = 0.01790*x* + 0.06491.

#### 3.2.3 Accuracy

Accuracy is degree of agreement of the test results generated by the method to the true value. In the optimization process, catechin target was found to be approximately 4 µg. Therefore, by following ICH guideline (ICH, 1994), the accuracy was assessed by percent recovery of standard catechin spiked amount to miang extract at 3.2, 4.0, and 4.8 μg, representing 80%, 100%, and 120% of the preliminary catechin (4.0 μg), respectively. Table 1 shows that the recovery was in the range of 98.84 and 103.53% with the RSD between 0.33% and 2.77%. However, this accuracy test was performed using contents lower than the middle point of the calibration (5 µg). Further analysis is required to cover the range of calibration. Nonetheless, the standard contents (2.5, 5.0, and 10.0 µg) were also evaluated to test the accuracy. The percent recovery was in the range of 96.90 and 105.00% with the percent RSD between 0.70% and 3.00% (Table 2). Percent recovery from both standard solution and spike solution was in the range of 95 and 105%, and therefore considered acceptable (AOAC International, 2016; ICH, 1994).

**TABLE 1:** Recovery studies of catechin at 80%, 100%, and 120% spiked

Spiked standard catechin (µg/spot)	Total height	Total catechin (μg)	Detected amount of added catechin (μg)	% Recovery (*n* = 3)	% RSD (*n* = 3)
0.0	0.1277	3.510	‐	‐	‐
3.2	0.1870	6.823	3.313	103.53	0.33
4.0	0.2012	7.617	4.107	102.67	2.26
4.8	0.2127	8.254	4.744	98.84	2.77

**TABLE 2:** Intra‐day and inter‐day precision studies of HPTLC method

Catechin (µg/μL)	% Recovery (*n* = 5)	Intra‐day precision (*n* = 5)		Inter‐day precision (*n* = 3 × 5)	
		*SD* (height)	% RSD	*SD* (height)	% RSD
0.25	96.90	0.0036	3.00	0.0057	4.94
0.50	100.16	0.0010	0.70	0.0029	1.93
1.00	105.00	0.0024	1.15	0.0076	3.52

#### 3.2.4 Precision

Precision is measurement of degree of repeatability of an analytical method under normal operation and is normally expressed as percent relative standard deviation (% RSD) for a statistically significant number of samples. Table 2 shows % RSD for intra‐day precision ranged from 0.70 to 3.00, while % RSD for inter‐day precision ranged from 1.93 to 4.94. The % RSD of intra‐day precision and inter‐day precision was lower than 3.70 and 6.00, respectively, and therefore considered acceptable (AOAC International, 2016; ICH, 1994).

#### 3.2.5 Limit of detection and limit of quantification

Limit of detection (LOD) is the lowest analyte amount in a sample that can be detected but not necessarily quantitated under stated experimental conditions. Limit of quantification (LOQ) is the lowest analyte amount in a sample that can be determined with acceptable precision and accuracy under stated experimental conditions. Catechin LOD was 0.78 μg, while LOQ was 2.37 μg, calculated from *SD* of y‐intercept as 0.0045 (*n* = 6) and mean slope of calibrations as 0.0188 (*n* = 6).

Collectively, the HPTLC with a developing solvent consisting of toluene: ethyl acetate: acetone: formic acid (6:6:6:1 v/v/v/v) exhibited acceptable specificity, linearity, accuracy, and precision to catechin in miang extracts according to ICH and AOAC. The validated HPTLC method is a powerful tool that could be used to ensure batch‐to‐batch reproducibility. It is also rapid and cost‐effective, and therefore can be used for regular evaluation during miang extraction. Although LOD and LOQ presented here indicate an adequate sensitivity (Kamboj & Saluja, 2017), HPTLC of *Acacia catechu* extract (Bhardwaj et al., 2020) and HPTLC of Kangra Tea (*C. sinensis*) (Kumar et al., 2016) showed catechin LOD and LOQ at nanogram. This suggests that method conditions and sample types may affect the observed sensitivity. Therefore, the HPTLC in this research should be further optimized for better analysis of miang catechin.

### 3.3 Determination of catechin contents in miang crude extract and semi‐purified fractions

**FIGURE 7:**
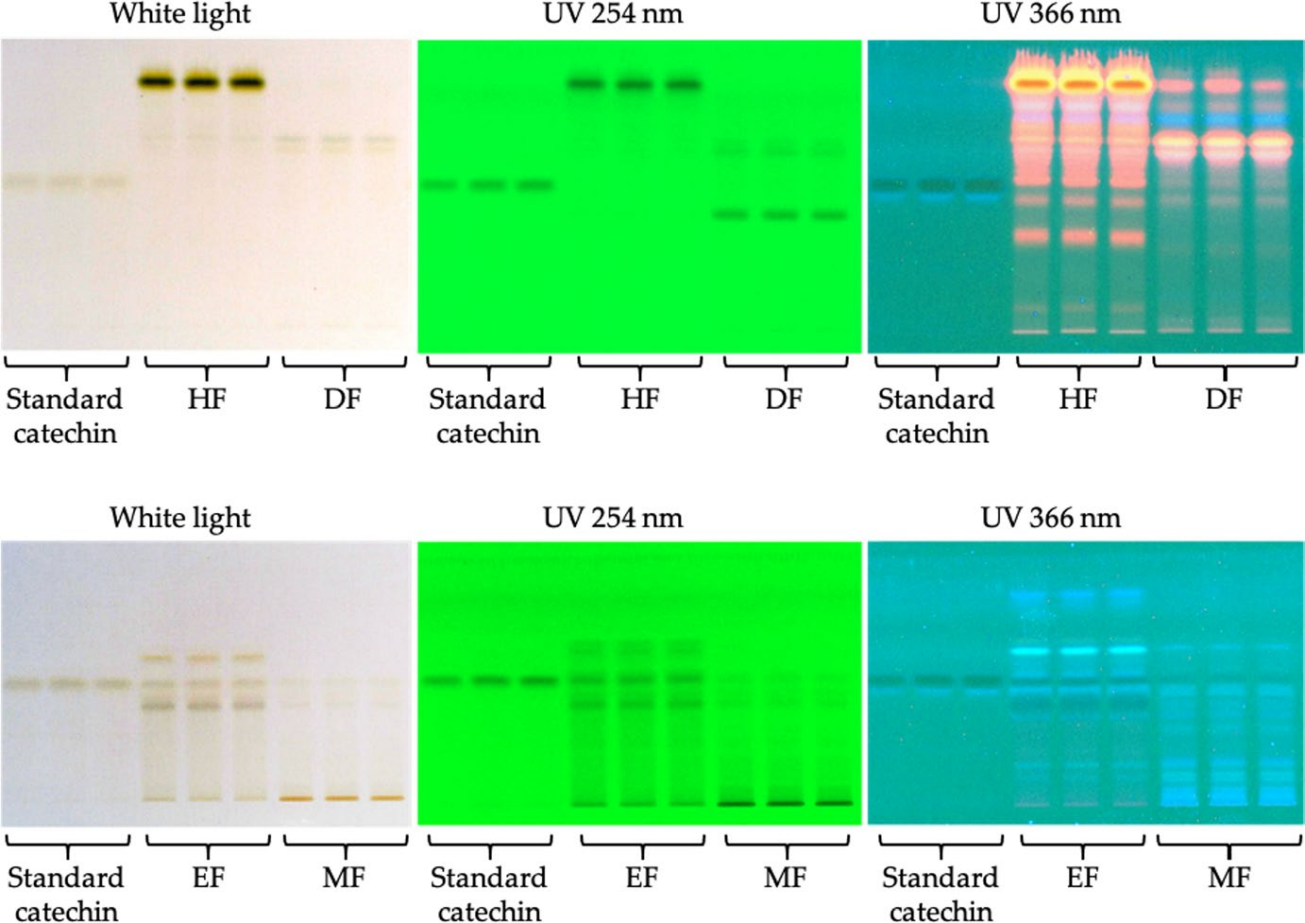
High‐performance thin‐layer chromatography (HPTLC) profiles of standard catechin, miang HF (hexane fraction), DF (dichloromethane fraction), EF (ethyl acetate fraction), and MF (methanol fraction) by using a developing solvent of toluene: ethyl acetate: acetone: formic acid (6:6:6:1 v/v/v/v) and measuring at white light, UV 254 nm, and UV 366 nm.

The HPTLC method was applied to determine catechin from miang extracts by a densitometric method (Table 3; Figures 5 and 7). EF had the highest catechin (25.78 ± 0.53%) which was higher than that from CE by two times (*p* < .0001) and MF by twenty‐eight times (*p* < .0001). Catechin in either HF or DF was not detectable. This suggests that ethyl acetate could either be a solvent choice by itself or be combined with others to isolate catechin‐enriched fraction. The applications of ethyl acetate for extracting tea catechins were previously demonstrated by several studies. A partitioning by water/ethyl acetate showed a higher refined purification of other catechins (EGCG, EGC, ECG, and EC) (Row & Jin, 2006). Total concentration of catechins was significantly higher in extracts of ethyl acetate and n‐hexane than that of n‐butanol following the extraction by water at 80°C (Dong et al., 2011). However, pressurized liquid extraction using methanol produced better recoveries of catechin (C) and epicatechin (EC) than water, ethanol, and ethyl acetate (Piñeiro et al., 2004). This indicates that ethyl acetate usage has a limitation depending on catechins of interest along with types and conditions of extraction methods (Koch et al., 2020). However, in this research, ethyl acetate is suggested to be the best solvent for isolating the catechin‐enriched fraction from CE by partitioning technique at room temperature.

**TABLE 3:** Catechin content analysis of miang crude extract and semi‐purified fractions

**Extracts**	**Extract amount (μg)**	**Catechin content (μg)**	**% Catechin content ± *SD***
CE	40	5.22	13.05 ± 0.91
HF^i^	320	ND	ND
DF^ii^	320	ND	ND
EF^iii^	20	5.16	25.78 ± 0.53
MF^iv^	320	2.93	0.92 ± 0.19

^i‐iv^CE (crude extract) was successively partitioned in indicated order using separatory funnels to produce HF (hexane fraction), DF (dichloromethane fraction), EF (ethyl acetate fraction), and MF (methanol fraction). CE (10 ± 1.17 g) was used for the solvent–solvent partitioning. ND indicates not detected.

### 3.4 Catechin stability profiles of miang crude extract and ethyl acetate fraction

**FIGURE 8:**
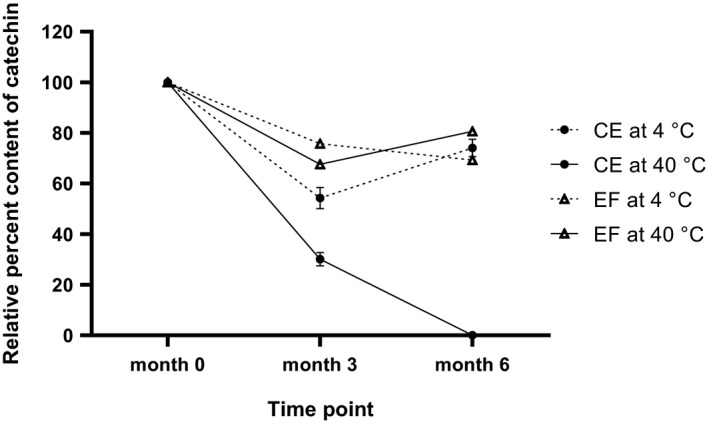
Stability profiles of catechin contents from miang CE (crude extract) and EF (ethyl acetate fraction). The extracts were studied under the accelerated conditions at 4°C and 40°C for 0, 3, and 6 months. All data are presented as mean ± *SD* of triplicate.

Several factors affect catechin stability including pH, temperature, oxygen, antioxidants, metal ions, and concentrations of other ingredients in tea extracts including catechins (Chen et al., 2001; Labbé et al., 2008; Piñeiro et al., 2004; Sang et al., 2005). The HPTLC method was applied to assess catechin (C) stability in EF compared to that in CE when stored without adding any preservative or antioxidants under accelerated condition (4°C and 40°C) for 0, 3, and 6 months. Figure 8 shows that catechin of both extracts kept at 4°C gradually decreased by approximately 30% and was not significantly different at 6 months (*p* = .099). However, at 40°C, the catechin in CE dramatically dropped by 70% at 3 months and was undetectable at 6 months. This points out that CE’s catechin degradation rate was slower at lower temperature, which is in accordance with a previous report that the degradation kinetics are affected by temperature and relative humidity (Li et al., 2011). Catechin stored at 40°C was significantly more stable in EF than CE at 3 months and 6 months (*p* < .001 and *p* < .0001, respectively). Partitioning of CE by ethyl acetate solvent resulted in similar catechin stability at 40°C and 4°C (approximately 80% and 70%, respectively), implying that EF could be stored at either 40°C or 4°C with little difference in the remaining percent catechin (*p* = .002).

**FIGURE 9:**
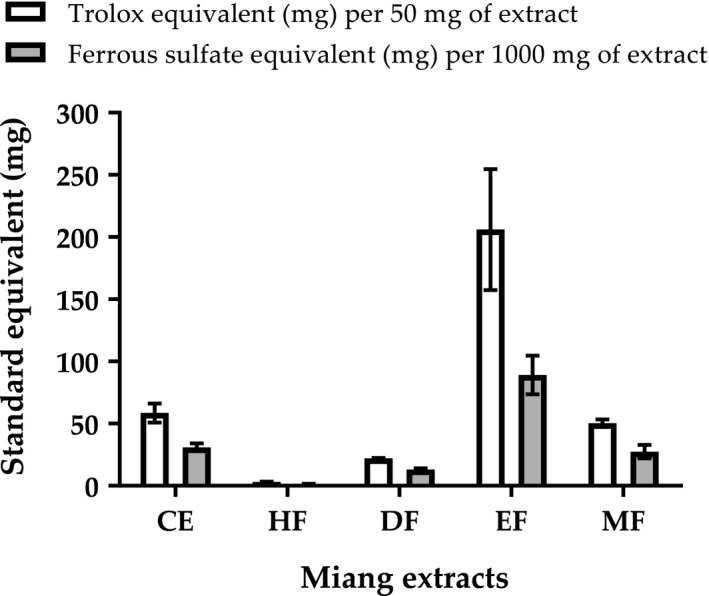
Antioxidant activities of miang CE (crude extract), HF (hexane fraction), DF (dichloromethane fraction), EF (ethyl acetate fraction), and MF (methanol fraction) expressed as Trolox equivalents by ABTS assay and ferrous sulfate equivalents by FRAP assay. All data are presented as mean ± *SD* of triplicate.

EF was not a purified extract but a semi‐purified fraction of CE. Therefore, the increased catechin stability in EF might be explained by the fraction possessing some characteristics that were different from CE. Catechins were more stable as their concentrations increased in solutions (Li et al., 2012). Therefore, the higher catechin (C) in EF than that of CE may be a factor contributing to its own increased stability. Additionally, higher catechin concentration could extend the shelf life of EGCG and other catechins (Sang et al., 2005). EF is enriched with catechin (C) but is not a pure catechin extract, so the stability of the catechin (C) could be potentially due to the presence of other catechins. However, further experiments are required to profile other catechins (EGCG, EGC, ECG, and EC) to investigate the impacts of the concentration relationship between each of the catechins on the stability of miang extracts (Labbé et al., 2008; Sang et al., 2005). Finally, the increased catechin (C) stability in EF could be due to the concentrations of other compounds that either were increased by being simultaneously isolated along with catechin (C) by ethyl acetate from CE or were decreased by being partitioned by other solvents during solvent–solvent partitioning. EF had the highest antioxidant potentials (Table 4; Figure 9). Therefore, the concentrated antioxidants may influence slower rate of catechin degradation in EF than that in CE by preventing or delaying auto‐oxidations during storage. The addition of ascorbic acid to tea extracts exerted dual functions on EGCG stability, where a low ascorbic acid concentration protected the degradation, but a high concentration promoted (Chen et al., 2021). Therefore, one of unknown compounds contributing to catechin (C) stability in EF could be ascorbic acid. Vitamin C in unbrewed leaves ranged from less than 3–178 mg/100g. Vitamin C exceeding 250 mg/100g was also found in unbrewed leaves of Japanese green tea (Somanchi et al., 2017). However, the effects of endogenous ascorbic acid on the catechin stability have not been investigated yet. Besides, ascorbic biosynthesis‐related gene expressions and content can vary by developmental stages, plant varieties, and temperature conditions (Li et al., 2016, 2017). Further investigations of other catechins and ascorbic acid could help creating approaches for improving both storage strategies and extraction processes to achieve and maintain high‐quality miang extracts.

**TABLE 4:** Antioxidant activities of miang crude extract and semi‐purified fractions

**Extracts**	**ABTS**	**Lipid peroxidation**	
	**IC_50_ (mg/mL)**	**% inhibition**	**IC_50_ (mg/mL)**
CE	10.63 ± 1.29^c^	20.83 ± 0.87^b^	6.08 ± 0.56^a^
HF^i^	20.55 ± 1.92 ^b^	ND	ND
DF^ii^	29.40 ± 0.30^a^	14.53 ± 0.87^c^	7.05 ± 0.64^a^
EF^iii^	3.32 ± 0.74^d^	27.62 ± 2.79^a^	4.36 ± 0.67^b^
MF^iv^	11.60 ± 0.66^c^	ND	ND

^i‐iv^CE (crude extract) was successively partitioned in indicated order using separatory funnels to produce HF (hexane fraction), DF (dichloromethane fraction), EF (ethyl acetate fraction), and MF (methanol fraction). CE (10 ± 1.17 g) was used for the solvent–solvent partitioning. All data are presented as mean ± *SD* of triplicate. ND indicates not detected. Superscript letters (a, b, c, and d) within the same column indicate significant differences of means between the groups based on Tukey's HSD one‐way ANOVA (*p* < .05).

### 3.5 Antioxidation activities of miang crude extract and semi‐purified fractions

Miang extracts possessed several phytochemicals (Figures 5 and 7). Each may exert antioxidant activities via different mechanisms. Therefore, to evaluate antioxidant potentials, miang extracts were assessed by ABTS, FRAP, and lipid peroxidation assays. In ABTS assay, Trolox equivalents (mg) were calculated to express free radical scavenging activities of miang extracts (50 mg). In FRAP assay, ferrous sulfate equivalents (mg) were calculated to express total reducing capacities of miang extracts (1,000 mg). Figure 9 shows that the activity patterns from ABTS and FRAP results were similar, suggesting a good agreement between the two assays. Therefore, miang extracts may exhibit antioxidant activities via both mechanisms. Although the mechanisms investigated by these assays are different, the results were comparative because the redox potential of Fe (III)‐TPTZ is comparable with that of ABTS^•+^ (Prior et al., 2005). Even though FRAP assay was conducted on extract amount twenty times higher than that of ABTS assay, FRAP results were lower than ABTS results in all extracts. This observation is in accordance with several studies where ABTS and FRAP assay may give comparable relative values; however, FRAP values are usually lower than ABTS values for a given series of antioxidant compounds (Prior et al., 2005). CE possessed a comparative antioxidant activity to MF (*p* > .05). HF and DF exhibited considerably lower antioxidant activities than MF, EF, and CE. Compounds isolated by hexane or dichloromethane have lower polarities than that of ethyl acetate and methanol. Therefore, low antioxidant activities in both HF and DF could be partly explained by the experimental conditions of ABTS and FRAP assays with low polar compounds. However, MF containing highest polar compounds exhibited lower activities than EF by four times and three times in ABTS and FRAP assays, respectively. Notably, EF exerted the highest antioxidant activities (*p* < .05) in all three assays. Owning to natural characteristics of phenolic compounds, catechins are well soluble in polar solvents. Therefore, the antioxidant activities of catechins are expected to be detected by ABTS and FRAP assays. Besides, EF’s richest catechin could potentially be responsible for most of the antioxidant activities determined by both assays. However, correlations between other catechins and ABTS and FRAP results are required to be further investigated. Taken together, this result supports previously reported catechin antioxidant efficacy exerting via both scavenging free radicals and preventing reactive oxygen species (ROS) generation by iron binding (Bernatoniene & Kopustinskiene, 2018). In lipid peroxidation assay, EF showed the highest inhibitory activity (27.62 ± 2.79%) which was higher than that of DF by two times (Table 4). This was not likely caused by catechins because phenolic solubility is not compatible with the test system. Potential explanation could be ethyl acetate sequestering non‐phenolic lipid peroxidation inhibitors as previously reported in green tea pheophytins a and b exerting inhibitions against linoleic acid peroxidation (Higashi‐Okai et al., 2000). Therefore, EF may provide attractive antioxidants with different polarities as well as different mechanisms.

## 4 CONCLUSION

The HPTLC for catechin analysis in miang extracts by a developing solvent consisting of toluene: ethyl acetate: acetone: formic acid (6:6:6:1 v/v/v/v) was validated according to ICH and AOAC guidelines. Semi‐purifications by ethyl acetate indicate improvements of catechin yield and stability as well as antioxidant activities. Therefore, EF is suggested to be the most attractive ingredient for product development. However, the stability should be further monitored beyond six months. The HPTLC method could potentially enable the usages of miang as a novel source or an alternative to unfermented tea in nutraceuticals and cosmeceuticals. Other applications may include selecting raw materials, optimizing extractions, assessing storage strategies, and assuring good agricultural practice (GAP). Further studies might be to compare catechins from tea plants of different growing conditions and harvesting seasons. Further developing HPTLC that simultaneously determines both catechins and caffeine could also provide a practical analytical tool for miang standardization according to European Pharmacopoeia (Council of Europe, 2018).

## CONFLICT OF INTEREST

We declare no conflicts of interest.

## **AUTHOR** **CONTRIBUTIONS**

P.T., J.J., and S.S.: involved in conceptualization, data curation, investigation, and methodology; J.J. and S.S. involved in validation; P.T., J.J., S.S., and R.P. involved in formal analysis; P.T., J.J., S.S., A.R., and H.P. involved in resources; P.T. and J.J. wrote the original draft and wrote, reviewed, and edited; P.T., J.J., and R.P. involved in visualization; J.J. and S.S. involved in supervision; S.S involved in project administration; P.T., J.J., S.S., R.P., A.R., and H.P involved in funding acquisition. All authors have read and agreed to the published version of the manuscript.

## ETHICAL APPROVAL

Our research did not contain any animal experiments and human subjects.

## References

[fsn32285-bib-0001] Alam, P. , Ezzeldin, E. , Iqbal, M. , Mostafa, G. A. E. , Anwer, M. K. , & Alqarni, M. H. (2020). Determination of delafloxacin in pharmaceutical formulations using a green RP‐HPTLC and NP‐HPTLC methods: A comparative study. Antibiotics, 9(6), 359. 10.3390/antibiotics9060359 PMC7344820 32630451

[fsn32285-bib-0002] Alqarni, M. H. , Alam, P. , Foudah, A. I. , Muharram, M. M. , & Shakeel, F. (2021). Combining normal/reversed‐phase HPTLC with univariate calibration for the piperine quantification with traditional and ultrasound‐assisted extracts of various food spices of *Piper nigrum* L. under green analytical chemistry viewpoint. Molecules, 26(3), 732. 33572524 10.3390/molecules26030732 PMC7866824

[fsn32285-bib-0003] AOAC International . (2016). Guidelines for Standard Method Performance Requirements. AOAC Official Methods of Analysis. Retrieved from http://www.eoma.aoac.org/app_f. pdf

[fsn32285-bib-0004] Attimarad, M. , Mueen Ahmed, K. K. , Aldhubaib, B. E. , & Harsha, S. (2011). High‐performance thin layer chromatography: A powerful analytical technique in pharmaceutical drug discovery. Pharm Methods., 2(2), 71–75. 23781433 10.4103/2229-4708.84436 PMC3658041

[fsn32285-bib-0005] Benzie, I. , & Strain, J. (1996). The ferric reducing ability of plasma (FRAP) as a measure of "antioxidant power": The FRAP assay. Analytical Biochemistry, 239, 70–76. 8660627 10.1006/abio.1996.0292

[fsn32285-bib-0006] Bernatoniene, J. , & Kopustinskiene, D. M. (2018). The role of catechins in cellular responses to oxidative stress. Molecules, 23(4), 965. 10.3390/molecules23040965 PMC6017297 29677167

[fsn32285-bib-0007] Bhardwaj, P. , Banarjee, A. , Jindal, D. , Kaur, C. , Singh, G. , Kumar, P. , Sharma, A. , & Kumar, R. (2020). Validation of TLC‐densitometry method for estimation of catechin in acacia catechu heartwood. Pharmaceutical Chemistry Journal, 54, 184–189.

[fsn32285-bib-0008] Chaikaew, S. , Baipong, S. , Sone, T. , Kanpiengjai, A. , Chui‐Chai, N. , Asano, K. , & Khanongnuch, C. (2017). Diversity of lactic acid bacteria from miang, a traditional fermented tea leaf in northern Thailand and their tannin‐tolerant ability in tea extract. Journal of Microbiology, 55(9), 720–729. 10.1007/s12275-017-7195-8 28865074

[fsn32285-bib-0009] Chen, L. , Wang, W. , Zhang, J. , Cui, H. , Ni, D. , & Jiang, H. (2021). Dual effects of ascorbic acid on the stability of EGCG by the oxidation product dehydroascorbic acid promoting the oxidation and inhibiting the hydrolysis pathway. Food Chemistry, 337, 127639. 32799157 10.1016/j.foodchem.2020.127639

[fsn32285-bib-0010] Chen, Z. Y. , Zhu, Q. Y. , Tsang, D. , & Huang, Y. (2001). Degradation of green tea catechins in tea drinks. Journal of Agriculture and Food Chemistry, 49(1), 477–482. 10.1021/jf000877h 11170614

[fsn32285-bib-0011] Chun, O. K. , Chung, S. J. , & Song, W. O. (2007). Estimated dietary flavonoid intake and major food sources of US adults. Journal of Nutrition, 137(5), 1244–1252. 10.1093/jn/137.5.1244 17449588

[fsn32285-bib-0012] Council of Europe . (2018). Green tea (Camelliae sinensis non fermentata folia). European Pharmacopoeia (9th ed.). Council of Europe.

[fsn32285-bib-0013] Dalluge, J. J. , & Nelson, B. C. (2000). Determination of tea catechins. Journal of Chromatography A, 881(1–2), 411–424. 10905724 10.1016/s0021-9673(00)00062-5

[fsn32285-bib-0014] Dong, J. J. , Ye, J. H. , Lu, J. L. , Zheng, X. Q. , & Liang, Y. R. (2011). Isolation of antioxidant catechins from green tea and its decaffeination. Food and Bioproducts Processing, 89(1), 62–66.

[fsn32285-bib-0015] Emran, T. B. , Rahman, M. A. , Uddin, M. M. , Rahman, M. M. , Uddin, M. Z. , Dash, R. , & Layzu, C. (2015). Effects of organic extracts and their different fractions of five Bangladeshi plants on in vitro thrombolysis. BMC Complementary and Alternative Medicine, 15, 128. 25902818 10.1186/s12906-015-0643-2 PMC4414290

[fsn32285-bib-0016] Foudah, A. I. , Alam, P. , Anwer, M. , Yusufoglu, H. S. , Abdel‐Kader, M. S. , & Shakeel, F. (2020). A green RP‐HPTLC‐densitometry method for the determination of diosmin in pharmaceutical formulations. Processes, 8(7), 817.

[fsn32285-bib-0017] Frankel, E. N. , & Neff, W. E. (1983). Formation of malondialdehyde from lipid oxidation products. Biochimica et Biophysica Acta, 754, 264–270.

[fsn32285-bib-0018] Grzesik, M. , Naparło, K. , Bartosz, G. , & Sadowska‐Bartosz, I. (2018). Antioxidant properties of catechins: Comparison with other antioxidants. Food Chemistry, 241, 480–492. 28958556 10.1016/j.foodchem.2017.08.117

[fsn32285-bib-0019] Higashi‐Okai, K. , Taniguchi, M. , & Okai, Y. (2000). Potent antioxidative activity of non‐polyphenolic fraction of green tea (Camellia sinensis) – Association with pheophytins a and b. Journal of the Science of Food and Agriculture, 80(1), 117–120.

[fsn32285-bib-0020] Higdon, J. V. , & Frei, B. (2003). Tea catechins and polyphenols: Health effects, metabolism, and antioxidant functions. Critical Reviews in Food Science and Nutrition, 43(1), 89–143. 12587987 10.1080/10408690390826464

[fsn32285-bib-0021] ICH . (1994). ICH Q2A: Validation of analytical procedures: text and methodology. Retrieved from https://database.ich.org/sites/default/files/Q2%28R1%9%0Guideline.pdf

[fsn32285-bib-0022] ICH . (2003). ICH Q1A(R2): Stability testing of new drug substances and products. Retrieved from https://database.ich.org/sites/default/files/Q1A%28R2%9%0Guideline.pdf

[fsn32285-bib-0023] Kamboj, A. , & Saluja, A. K. (2017). Development of validated HPTLC method for quantification of stigmasterol from leaf and stem of *Bryophyllum pinnatum* . Arabian Journal of Chemistry, 10, S2644–S2650.

[fsn32285-bib-0024] Khanongnuch, C. , Unban, K. , Kanpiengjai, A. , & Saenjum, C. (2017). Recent research advances and ethno‐botanical history of miang, a traditional fermented tea (*Camellia sinensis* var. *assamica*) of northern Thailand. Journal of Ethnic Foods, 4(3), 135–144.

[fsn32285-bib-0025] Klayraung, S. , & Okonogi, S. (2009). Antibacterial and antioxidant activities of acid and bile resistant strains of *Lactobacillus fermentum* isolated from miang. Brazilian Journal of Microbiology, 40(4), 757–766. 24031422 10.1590/S1517-83822009000400005 PMC3768562

[fsn32285-bib-0026] Koch, W. , Kukuła‐Koch, W. , Czop, M. , Helon, P. , & Gumbarewicz, E. (2020). The role of extracting solvents in the recovery of polyphenols from green tea and its antiradical activity supported by principal component analysis. Molecules, 25(9), 2173. 10.3390/molecules25092173 PMC7248709 32384780

[fsn32285-bib-0027] Kumar, D. , Gulati, A. , & Sharma, U. (2016). Determination of theanine and catechin in *Camellia sinensis* (Kangra tea) leaves by HPTLC and NMR techniques. Food Analytical Methods, 9(6), 1666–1674.

[fsn32285-bib-0028] Labbé, D. , Têtu, B. , Trudel, D. , & Bazinet, L. (2008). Catechin stability of EGC‐and EGCG‐enriched tea drinks produced by a two‐step extraction procedure. Food Chemistry, 111(1), 139–143.

[fsn32285-bib-0029] Li, H. , Huang, W. , Wang, G. L. , Wang, W. L. , Cui, X. , & Zhuang, J. (2017). Transcriptomic analysis of the biosynthesis, recycling, and distribution of ascorbic acid during leaf development in tea plant (*Camellia sinensis* (L.). O. Kuntze). Scientific Reports, 7, 46212. 28393854 10.1038/srep46212 PMC5385563

[fsn32285-bib-0030] Li, H. , Huang, W. , Wang, G. L. , Wu, Z. J. , & Zhuang, J. (2016). Expression profile analysis of ascorbic acid‐related genes in response to temperature stress in the tea plant, *Camellia sinensis* (L.) O. Kuntze. Genetics and Molecular Research, 15, 1–10. 10.4238/gmr.15048756 27808374

[fsn32285-bib-0031] Li, N. , Taylor, L. S. , Ferruzzi, M. G. , & Mauer, L. J. (2012). Kinetic study of catechin stability: Effects of pH, concentration, and temperature. Journal of Agriculture and Food Chemistry, 60(51), 12531–12539. 10.1021/jf304116s 23205662

[fsn32285-bib-0032] Li, N. , Taylor, L. S. , & Mauer, L. J. (2011). Degradation kinetics of catechins in green tea powder: Effects of temperature and relative humidity. Journal of Agriculture and Food Chemistry, 59(11), 6082–6090. 10.1021/jf200203n 21495730

[fsn32285-bib-0033] Marinovic, M. P. , Morandi, A. C. , & Otton, R. (2015). Green tea catechins alone or in combination alter functional parameters of human neutrophils via suppressing the activation of TLR‐4/NFκB p65 signal pathway. Toxicology in Vitro, 29(7), 1766–1778. 26187476 10.1016/j.tiv.2015.07.014

[fsn32285-bib-0034] Matsumoto, K. , Yamada, H. , Takuma, N. , Niino, H. , & Sagesaka, Y. M. (2011). Effects of green tea catechins and theanine on preventing influenza infection among healthcare workers: A randomized controlled trial. BMC Complementary and Alternative Medicine, 11, 15. 21338496 10.1186/1472-6882-11-15 PMC3049752

[fsn32285-bib-0035] Matsunaga, K. , Klein, T. W. , Friedman, H. , & Yamamoto, Y. (2001). Legionella pneumophila replication in macrophages inhibited by selective immunomodulatory effects on cytokine formation by epigallocatechin gallate, a major form of tea catechins. Infection and Immunity, 69(6), 3947–3953. 11349063 10.1128/IAI.69.6.3947-3953.2001 PMC98432

[fsn32285-bib-0036] Mukherjee, P. K. (2002). Problems and prospects for good manufacturing practice for herbal drugs in Indian systems of medicine. Therapeutic Innovation & Regulatory Science, 36(3), 635–644.

[fsn32285-bib-0037] Murase, T. , Nagasawa, A. , Suzuki, J. , Hase, T. , & Tokimitsu, I. (2002). Beneficial effects of tea catechins on diet‐induced obesity: Stimulation of lipid catabolism in the liver. International Journal of Obesity and Related Metabolic Disorders, 26(11), 1459–1464. 12439647 10.1038/sj.ijo.0802141

[fsn32285-bib-0038] Nagao, T. , Hase, T. , & Tokimitsu, I. (2007). A green tea extract high in catechins reduces body fat and cardiovascular risks in humans. Obesity, 15(6), 1473–1483. 17557985 10.1038/oby.2007.176

[fsn32285-bib-0039] Piñeiro, Z. , Palma, M. , & Barroso, C. G. (2004). Determination of catechins by means of extraction with pressurized liquids. Journal of Chromatography A, 1026(1), 19–23. 14763728 10.1016/j.chroma.2003.10.096

[fsn32285-bib-0040] Prior, R. L. , Wu, X. , & Schaich, K. (2005). Standardized methods for the determination of antioxidant capacity and phenolics in foods and dietary supplements. Journal of Agriculture and Food Chemistry, 53(10), 4290–4302. 10.1021/jf0502698 15884874

[fsn32285-bib-0041] Row, K. H. , & Jin, Y. (2006). Recovery of catechin compounds from Korean tea by solvent extraction. Bioresource Technology, 97(5), 790–793. 15919205 10.1016/j.biortech.2005.04.001

[fsn32285-bib-0042] Saenjum, C. , Chaiyasut, C. , Kadchumsang, S. , Chansakaow, S. , & Suttajit, M. (2010). Antioxidant activity and protective effects on DNA damage of *Caesalpinia sappan* L. extract. Journal of Medicinal Plants Research, 4, 1594–1608.

[fsn32285-bib-0043] Sang, S. , Lee, M. J. , Hou, Z. , Ho, C. T. , & Yang, C. S. (2005). Stability of tea polyphenol (−)‐epigallocatechin‐3‐gallate and formation of dimers and epimers under common experimental conditions. Journal of Agriculture and Food Chemistry, 53(24), 9478–9484. 10.1021/jf0519055 16302765

[fsn32285-bib-0044] Singh, B. N. , Shankar, S. , & Srivastava, R. K. (2011). Green tea catechin, epigallocatechin‐3‐gallate (EGCG): Mechanisms, perspectives and clinical applications. Biochemical Pharmacology, 82(12), 1807–1821. 21827739 10.1016/j.bcp.2011.07.093 PMC4082721

[fsn32285-bib-0045] Somanchi, M. , Phillips, K. , Haile, E. , & Pehrsson, P. (2017). Vitamin C content in dried and brewed green tea from the US retail market. The FASEB Journal, 31(1_supplement), 956–958.

[fsn32285-bib-0046] Tanasupawat, S. , Pakdeeto, A. , Thawai, C. , Yukphan, P. , & Okada, S. (2007). Identification of lactic acid bacteria from fermented tea leaves (miang) in Thailand and proposals of *Lactobacillus thailandensis* sp. nov., *Lactobacillus camelliae* sp. nov., and *Pediococcus siamensis* sp. nov. Journal of General and Applied Microbiology, 53(1), 7–15. 10.2323/jgam.53.7 17429157

[fsn32285-bib-0047] Tran, J. (2013). Green tea: A potential alternative anti‐infectious agent catechins and viral infections. Advances in Anthropology, 3, 198–202.

[fsn32285-bib-0048] Unban, K. , Khatthongngam, N. , Shetty, K. , & Khanongnuch, C. (2019). Nutritional biotransformation in traditional fermented tea (Miang) from north Thailand and its impact on antioxidant and antimicrobial activities. Journal of Food Science and Technology, 56(5), 2687–2699. 31168151 10.1007/s13197-019-03758-x PMC6525707

[fsn32285-bib-0049] Upton, R. T. (2010). Use of high‐performance thin layer chromatography by the American Herbal Pharmacopoeia. Journal of AOAC International, 93(5), 1349–1354. 21140643

[fsn32285-bib-0050] Vogiatzoglou, A. , Mulligan, A. A. , Lentjes, M. A. , Luben, R. N. , Spencer, J. P. , Schroeter, H. , Khaw, K. T. , & Kuhnle, G. G. (2015). Flavonoid intake in European adults (18 to 64 years). PLoS One, 10(5), e0128132. 26010916 10.1371/journal.pone.0128132 PMC4444122

[fsn32285-bib-0051] Wang, H. , Provan, G. J. , & Helliwell, K. (2000). Tea flavonoids: Their functions, utilisation and analysis. Trends in Food Science & Technology, 11(4–5), 152–160.

[fsn32285-bib-0052] Youn, H. S. , Lee, J. Y. , Saitoh, S. I. , Miyake, K. , Kang, K. W. , Choi, Y. J. , & Hwang, D. H. (2006). Suppression of MyD88‐and TRIF‐dependent signaling pathways of Toll‐like receptor by (−)‐epigallocatechin‐3‐gallate, a polyphenol component of green tea. Biochemical Pharmacology, 72(7), 850–859. 16890209 10.1016/j.bcp.2006.06.021

[fsn32285-bib-0053] Zuo, Y. , Chen, H. , & Deng, Y. (2002). Simultaneous determination of catechins, caffeine and gallic acids in green, Oolong, black and pu‐erh teas using HPLC with a photodiode array detector. Talanta, 57(2), 307–316. 18968631 10.1016/s0039-9140(02)00030-9

